# Air Travel Concerns and Complaints of Persons Living With Dementia and Their Travel Companions

**DOI:** 10.1093/geroni/igaa057.371

**Published:** 2020-12-16

**Authors:** Colleen Peterson, Tamara Statz, Sara Barsel, Robyn Birkeland, Joseph Gaugler, Jessica Finlay

**Affiliations:** 1 University of Minnesota, Saint Paul, Minnesota, United States; 2 University of Minnesota, Minneapolis, Minnesota, United States; 3 Roseville Alzheimer’s & Dementia Community Action Team, Minneapolis, Minnesota, United States; 4 University of Michigan, Ann Arbor, Michigan, United States

## Abstract

Air travel is increasingly accessible and rising numbers of older adults choose to fly. The prevalence of Alzheimer’s disease and related dementias (ADRD) is also expected to increase due to advances in longevity and the aging Baby Boomer population. An ADRD diagnosis does not necessarily end the desire to travel for leisure, and air travel may be necessary for family and/or care reasons. Taken together, more persons living with ADRD are expected to use air travel. This is concerning since adverse outcomes, including confusion and delirium, can be exacerbated by unfamiliar environments, such as airports and in-flight situations. Yet there is little research on air travel with this population. The current study seeks to 1) understand air travel experiences, issues, and needs of persons living with ADRD and their travel companions; and 2) identify facilitators and barriers to safe and comfortable air travel for persons living with ADRD. Persons living with ADRD (n=49)and ADRD travel companions (n=176) provided information on their air travel experiences through a nationally distributed web-survey. They traveled for family visits (80.0%) and leisure (38.0%). Both persons living with ADRD and travel companions indicated anxiety (50% and 60.7%, respectively), difficulty understanding announcements and signage (39.1%, 55.2%) and getting lost or separated (37.5%, 53.2%) as primary travel concerns. Qualitative comments identified themes of frustration with locating family restrooms and quiet spaces. Results from this study will inform ongoing efforts to develop dementia-friendly airports and improve older adult travel experiences, independence, and quality of life.

